# The impact of maternal health insurance coverage and adequate healthcare services utilisation on the risk of under-five mortality in Nigeria: a cross-sectional study

**DOI:** 10.1186/s13690-022-00968-2

**Published:** 2022-09-13

**Authors:** Chukwuechefulam Kingsley Imo, Nicole De Wet-Billings, Uche Charlie Isiugo-Abanihe

**Affiliations:** 1 Department of Sociology, Adekunle Ajasin University, Akoko-Akungba, Ondo State Nigeria; 2 Demography and Population Studies, University of the Witwatersrand, Johannesburg, South Africa; 3 Department of Sociology, University of Ibadan, Ibadan, Oyo State Nigeria

**Keywords:** Health insurance, Healthcare services utilisation, Mothers, Under-five mortality, Nigeria

## Abstract

**Background:**

Despite the progress in reducing under-five mortality (U-5 M) in recent years, these deaths remain considerably high in Nigeria. This could be attributed to poor health policies including inequality of health insurance coverage and access to adequate healthcare services utilisations which has remained inimical to achieving sustainable development goals (SDGs). Therefore, this study examined the impact of maternal health insurance coverage and adequate healthcare services utilisation on the risk of U-5 M in Nigeria.

**Methods:**

The data for the study were derived from the 2018 Nigeria Demographic and Health Survey and comprised a weighted sample of 127,545 birth histories of childbearing women. Descriptive and analytical analyses were carried out, including frequency tables and multivariate using Cox proportional regression. The results were presented as hazard ratios (HR) with 95% confidence intervals (CIs). Data were analyzed using Stata software version 15.1.

**Results:**

The results showed that 14.3% of the sampled birth histories of the childbearing women were children who died before age 5. The results further showed that 97.7% of the children were of mothers who have health insurance and over one-half (56.5%) were children whose mothers had adequate healthcare services utilisation. The risk of under-five death was significantly lower among the children of mothers who were covered by health insurance (HR: 0.66, CI: 0.42–1.02) and those whose mothers utilised adequate healthcare services (HR: 0.78, CI: 0.68–0.90). A similar result was observed among children whose mothers reported that distance to the health facility was not a problem (HR: 0.81, CI: 0.72–0.86). Some mothers’ characteristics including educational attainment, wealth quintile and region of residence significantly influenced the risk of U-5 M.

**Conclusions:**

The study established that maternal health insurance coverage and adequate healthcare services utilisation were found to be protective factors against the risk of U-5 M. Also, the revealed low health insurance coverage of mothers calls for more pragmatic policy and intervention programmes through health insurance to achieve SDGs targets of ending preventable deaths of children under 5 years of age and ensuring quality, as well as universal access to maternal and child healthcare services.

## Background

Globally, adequate healthcare service during pregnancy, childbirth and post-delivery periods is of great importance for the survival and well-being of mothers and children. In sub-Saharan Africa (SSA), which Nigeria is an integral part of, it is revealed that most preventable causes of deaths related to pregnancy and childbirth due to inadequate healthcare services utilisation affect children’s health outcomes [1]. Long distance to health facilities, poor socioeconomic factors, lack of skilled workers and facilities, and high financial burden on families of expectant mothers contribute to inadequate healthcare services patronage in Nigeria and some countries with similar characteristics [2–4]. In SSA countries, access to universal health insurance for all people is the major health-system policy focus. According to World Health Organisation (WHO), universal health coverage is based on the fact that everyone can obtain the needed healthcare services at high quality, irrespective of social inequality by providing financial protection from the costs of using health services in the country [5]. In 2004, the Federal Government of Nigeria instituted the National Health Insurance Scheme (NHIS) which can be obtained from private organisations or government agencies. The scheme is to improve the health of all Nigerians at an affordable cost, especially mothers during antenatal, delivery and postnatal care, as well as all live births during the post-natal period of 12 weeks from the date of delivery [6, 7]. As expected, health insurance reduces high out-of-pocket expenditures and improves health-seeking and utilisation behaviours [8], but the inequality of coverage across socio-economic and employment lines directly or indirectly affect child health outcome in Nigeria.

Under-five mortality (U-5 M), defined as the number of children dying before the fifth birthday (0–59 months) has remained high in low-income countries with an average rate of 68 deaths per 1000 live births in 2019 as against 69 deaths per 1000 live births in 2017 [1]. Despite the progress in reducing U-5 M, these deaths have remained considerably high in Nigeria, the sixth highest in the world and the second in Africa [9]. In Nigeria, the U-5 M rate declined from 213 deaths per 1000 live births in 1990 to 132 deaths per 1000 live births in 2018, which implies that more than 1 in every 8 children in Nigeria dies before 5 years [10]. No doubt, the slow pace of decline in U-5 M could be attributed to poor health policies including inequality of health insurance coverage and access to quality healthcare services [11, 12].

There have been several studies on the risk factors of U-5 M in Nigeria. Some of these previous studies have investigated the risk factors of U-5 M including maternal socio-demographic factors and antenatal care utilization [13, 14], contextual factors [15, 16] and family type and ethnicity [17, 18]. Other studies identified housing materials [19], neighbourhood poverty and household use of solid fuel for cooking [20, 21], as well as dietary diversity, environment and health-related factors [22] as the risk factors for U-5 M in Nigeria. However, there is a paucity of empirical research in the literature examining the influence of maternal health insurance coverage and healthcare services utilisation on the risk of U5M. Understanding the influence of maternal health insurance coverage and adequate healthcare services utilisation on U5M is essential to the design and assessment of interventions to improve both maternal and child health. This is expected to provide up-to-date information, relevant policy and programmatic recommendations towards achieving sustainable development goals (SDGs) target of ending preventable deaths of newborns and children under the age of 5 years by ensuring universal health coverage and access to quality essential maternal and child healthcare services in Nigeria. Therefore, this study used the latest Nigeria Demographic and Health Survey to examine the influence of maternal health insurance coverage and adequate healthcare services utilisation on U5M in Nigeria.

### Theoretical framework

The Health Belief Model (HBM) was adopted as a theoretical framework in this study. The HBM postulates that certain constructs including risk susceptibility, risk severity, action benefits, barriers to action, self-efficacy, and cues to action predict health behaviour [23]. The model enables us to understand two aspects of women’s representations of health and behaviours including threat perception and health behavioural evaluation in reducing the risk of a negative health outcome [24]. Previous studies have adopted the model to evaluate the trends in utilisation of preventive healthcare, as well as visiting health facilities for maternal and child healthcare [25, 26]. Women’s threat perception and health behavioural evaluation prompt them to action relating to healthcare services utilisation during pregnancy and after childbirth [27]. Despite focusing on the individual, the model recognises and addresses the social context in which health behaviours take place [28]. This social context which includes health policies and strategies encourages access to healthcare services to reduce health risks [29]. Certain health policies and strategies, especially health insurance necessitate understanding regarding health-seeking behaviour for quality healthcare promotion and improved quality of life [30]. Health insurance creates an avenue and improves access to quality healthcare services which promotes positive maternal and child health outcomes [31]. Consequently, the cost of seeking healthcare services and knowledge of health complications during and after pregnancy might influence a woman’s perception of the severity and threats of health risks [32]. In addition, the use of healthcare services could be influenced by their availability and quality through health insurance, as well as the social structure and personal characteristics of the women [33, 34].

Knowledge of health complications and actual threats to both mother and child health are provided through health messages and these convince women that a particular behaviour can reduce their risks, hence encouraging a positive change in healthcare behaviour [35]. No doubt, even when healthcare services are publicly funded, health-related behaviour patterns are certainly associated with socioeconomic status. Therefore, the HBM proposed that internal and external cues to action could activate the women’s decision-making process for a health-promoting action [36]. This encourages mothers to seek medical attention for their sick children because they are convinced that the financial hardships that may result from large or unexpected medical bills are covered by health insurance [37, 38]. In this situation, women are more likely to engage in a specific health-seeking practice during pregnancy and after childbirth when perceived benefits override barriers [39], which have a positive influence on child survival, particularly children who are below the age of 5 years [7].

In the context of this study, perceived susceptibility means a high probability of proneness to the risk of child death. As a result, the perceived threat to the identified risk of child death and the information on the preventive measures motivate women to take preventative action provided the modifying factors are advantageous and favourable. These factors include the availability of financial support to help the action (health-seeking behaviour) at an affordable cost, as well as equal accessibility and distribution of the resources. With reference to HBM, this study hypothesized that women who are covered by health insurance tend to disregard the threat perception of seeking healthcare services and make health evaluations that are protective against the risks of U-5 M.

## Methods

### Data source

The data for this child-based study was obtained from the birth re-code data file of the 2018 Nigeria Demographic and Health Survey (NDHS). The survey is a cross-sectional study and the latest in the periodic Demographic and Health Survey (DHS) series, which started in Nigeria at the national level in 1990. Data were generated from 41,821 women aged 15–49 and 13,311 men aged 15–59. A detailed report of the data collection methods and procedures for 2018 NDHS has been published elsewhere [10]. The survey provides up-to-date information on demographic and socio-economic factors, health insurance coverage and other health indicators including childhood mortality and maternal mortality and maternal and child health in Nigeria. The detailed report of the methods and procedures adopted in the collection of data for 2018 NDHS has been published elsewhere [10]. The analyses for this study covered a weighted sample of 127,545 birth histories of childbearing women aged 15–49 years within 5 years before the survey (i.e. 2013–2018).

### Outcome variable

The outcome variable was under-five mortality (U-5 M) defined as the probability of a child dying between birth and exactly 5 years of age and expressed per 1000 live births [40]. For this study, this is measured as the duration of survival since birth in months and dichotomised into ‘yes’ (for children who died before age 5) and coded as 1, otherwise classified as ‘no’ (being alive) and coded as 0.

### Explanatory variables

The main explanatory variables were ‘health insurance coverage’ expressed as the insurance coverage that pays for medical expenses of an insured individual from government agencies or private organisations [5] and ‘adequate healthcare services’ utilisation defined as the essential services of quality of care and health services which underpins and is fundamental to universal health coverage [41]. The composite measure of adequate healthcare services utilisation was created from mothers’ responses to the four selected components of maternal and child health services utilisation. These include several antenatal care (ANC) visits during pregnancy, place of delivery, the person who performed the baby’s postnatal check within 2 months of delivery and the person who checked the respondent’s health before discharge. The responses for each level of healthcare service utilization were collapsed into two categories. Respondents who had at least 4 or more ANC visits, delivered in a health facility and those whose babies were checked by skilled health personnel were categorized as ‘adequate’ healthcare service utilization, otherwise classified as ‘inadequate’. Concerning the number of ANC visits, data ranged from ‘0’ to ‘20’ visits during the period of pregnancy with at least 4 visits considered for this study as having attended adequate ANC visits based on WHO’s standard at the time of the survey without prejudice to the recent WHO recommendation of a minimum of 8 visits [42, 43].

The covariates included maternal age, marital status, educational attainment, employment status, wealth index, place of residence, region, distance to the health facility and women’s decision-making autonomy relating to their healthcare and earnings are prerequisites for improvements in maternal and child health [44]. Women’s ability to attend to their health and utilize healthcare facilities appropriately may depend in part on their decision-making autonomy defined as the extent of women’s independence on finances, matters on their health and that of the households without having to obtain permission [45]. The selection of all the variables was informed by their documented significant association with healthcare services utilisation and child health outcome.

### Statistical analysis

Three different analyses (univariate, bivariate and multivariate) were carried out in this study. At the univariate level, descriptive statistics related to the characteristics of the study population were expressed as the total (see Table 1). Pearson chi-square test was used at the bivariate level in Table 2 to examine the association between health insurance coverage and healthcare services utilisation, while Table 3 investigates the relationship between the outcome variable (under-five mortality) and main explanatory variables, as well as the covariates. At the multivariate level in Table 4, Cox proportional regression analysis was used to examine the risk of U-5 M. The Cox regression procedure is considered appropriate for this study for its usefulness in the analysis of survival data and because it takes care of censoring problems in mortality data since some children are exposed to the risk of mortality [17, 46]. The results were presented as hazard ratios (HR) with 95% confidence intervals (CIs). Three models were fitted to examine the risk factors of U-5 M. Model 1 presents the adjusted HR showing the relationship between U-5 M and the main explanatory variables. In addition to the main explanatory variables, Model 2 adjusted for the effect of the mother’s characteristics. Model 3 adjusted for the significant mother’s characteristics in Model 2 and decision-making autonomy measures and place of residence (urban, rural and geopolitical zones). The dataset was carefully checked for missing values that were excluded from the analyses and weighted with the appropriate sampling weights as per the Demographic and Health Survey (DHS) sampling scheme before the analyses. All the analyses were conducted using Stata software (version 15.1).

**Table 1:** Percentage distribution of the study population, NDHS 2018

Variable	*n* (%)
**Under-five mortality**	
No	109,325(85.7)
Yes	18,220(14.3%)
**Maternal health insurance coverage**	
No	124,602(97.7)
Yes	2943(2.3)
**Healthcare service utilization**	
Inadequate	14,767(43.5)
Adequate	19,157(56.5)
**Maternal age (years)**	
15–24	10,004(7.8)
25–34	42,625(33.4)
35–49	74,916(58.7)
**Marital status**	
Never in union	1581(1.2)
Married/living with partner	117,150(91.9)
Widowed/divorced/separated	8814(6.9)
**Mother’s educational attainment**	
No education	63,699(50.0)
Primary	25,311(19.8)
Secondary or higher	38.535(30.2)
**Mother’s employment status**	
Not working	33,052(25.9)
currently working	94,493(74.1)
**Wealth quintile**	
Poor	60,596(47.5)
Middle	27,120(21.3)
Rich	39,829(31.2)
**Place of residence**	
Urban	44,111(34.6)
Rural	83,434(65.4)
**Region**	
North-central	21,656(17.0)
North-east	26,293(20.6)
North-west	39,928(31.3)
South-east	14,072(11.0)
South-south	12,436(9.8)
South-west	13,160(10.3)
**Distance to the health facility**	
Big problem	38,251(30.0)
Not a problem	89,294(70.0)
**Decision on respondent’s healthcare**	
Husband/partner and other	68,250(16.7)
Jointly	37,294(31.8)
Alone	11,606(9.9)
**Decision on how to spend respondent’s earnings**	
Husband/partner and other	7041(9.3)
Jointly	13,943(18.5)
Alone	54,407(72.2)

**Table 2:** Utilisation of healthcare services by health insurance coverage, NDHS 2018

	Inadequate	Adequate	Total	
	*N* = 14,767	*N* = 19,157	*N* = 33,924	
Variable	*n* (%)	*n* (%)	*n* (%)	*χ*^2^
**Health insurance coverage**				223.9***
No	14,646(44.1)	18.543(55.9)	33,189	
Yes	121(16.5)	614(83.5)	735	

***p < 0.001

**Table 3:** Maternal health insurance and healthcare service utilisation factors, as well as covariates associated with U-5 M, NDHS 2018

	Alive	Dead	Total	
	*N* = 109,325	*N* = 18,220	*N* = 127,545	
Variable	*n* (%)	*n* (%)	*n* (%)	*χ*^2^
**Maternal health insurance coverage**				34.0***
No	106,693(85.6)	17,909(14.4)	124,602(97.7)	
Yes	2632(89.4)	311(10.6)	2943(2.3)	
**Healthcare service utilization**				313.4***
Inadequate	12,896(87.3)	1871(12.7)	14,767(43.5)	
Adequate	17,817(93.0)	1340(7.0)	19,157(56.5)	
**Maternal age (years)**				294.4***
15–24	8865(88.6)	1139(11.4)	10,004(7.8)	
25–34	37,286(87.5)	5339(12.5)	42,625(33.4)	
35–49	63,174(84.3)	11,742(15.7)	74,916(58.7)	
**Marital status**				52.0***
Never in union	1440(91.1)	141(8.9)	1581(1.2)	
Married/living with partner	100,216(85.6)	16,934(14.4)	117,150(91.9)	
Widowed/divorced/separated	7669(87.0)	1145(13.0)	8814(6.9)	
**Mother’s educational attainment**				200.2***
No education	51,893(81.5)	11,806(18.5)	63,699(50.0)	
Primary	21,993(86.9)	3318(13.1)	25,311(19.8)	
Secondary or higher	35,439(92.0)	3096(8.0)	38.535(30.2)	
**Mother’s employment status**				135.5***
Not working	27.693(83.8)	5359(29.4)	33,052(25.9)	
Currently working	81,632(86.4)	12,861(13.6)	94,493(74.1)	
**Wealth quintile**				213.3***
Poor	49,318(81.4)	11,278(18.6)	60,596(47.5)	
Middle	23,575(86.9)	3545(13.1)	27,120(21.3)	
Rich	36,432(91.5)	3397(8.5)	39,829(31.2)	
**Place of residence**				814.4***
Urban	39,506(89.6)	4605(10.4)	44,111(34.6)	
Rural	69,819(83.7)	13,615(16.3)	83,434(65.4)	
**Region**				303.2***
North-central	19,243(88.9)	2413(11.1)	21,656(17.0)	
North-east	22,179(84.4)	4114(15.7)	26,293(20.6)	
North-west	31,418(78.7)	8510(21.3)	39,928(31.3)	
South-east	12,906(91.7)	1166(8.3)	14,072(11.0)	
South-south	11,425(91.9)	1011(8.1)	12,436(9.8)	
South-west	12,154(92.4)	1006(7.6)	13,160(10.3)	
**Distance to the health facility**				40.4***
Big problem	32,423(84.8)	5828(15.2)	38,251(30.0)	
Not a problem	76,902(86.1)	12,392(13.9)	89,294(70.0)	
**Decision on respondent’s healthcare**				642.5***
Husband/partner and other	56,889(83.4)	11,361(16.7)	68,250(16.7)	
Jointly	33,135(88.9)	4159(11.2)	37,294(31.8)	
Alone	10,192(87.8)	1414(12.2)	11,606(9.9)	
**Decision on how to spend respondent’s** **Earnings**				320.5***
Husband/partner and other	5996(85.2)	1045(14.8)	7041(9.3)	
Jointly	12,636(90.6)	1307(9.4)	13,943(18.5)	
Alone	46,102(84.7)	8305(15.3)	54,407(72.2)	

***p < 0.001

**Table 4:** Hazard ratio (HR) and 95% confidence interval (CI) for maternal health insurance, healthcare services utilisation and covariates associated with U-5 M, NDHS 2018

	Model 1	Model 2	Model 3
Variable	HR(95% CI)	HR(95% CI)	HR(95% CI)
**Health insurance coverage**			
No (Ref.)	1.00	1.00	1.00
Yes	0.62(0.46–0.88)**	0.75(0.54–1.04)*	0.66(0.42–1.02)*
**Healthcare service utilization**			
Inadequate (Ref.)	1.00	1.00	1.00
Adequate	0.56(0.51–0.62)***	0.73(0.66–0.81)***	0.78(0.68–0.90)**
**Distance to the health facility**			
Big problem (Ref.)		–	1.00
Not a problem		–	0.81(0.72–0.86)**
**Decision on respondent’s healthcare**			
Husband/partner and other (Ref.)		–	1.00
Jointly		–	0.97(0.84–1.11)
Alone		–	0.82(0.66–1.01)
**Decision on how to spend respondent’s** **Earnings**			
Husband/partner and other (Ref.)		–	1.00
Jointly		–	0.91(0.71–1.17)
Alone		–	1.17(0.98–1.41)
**Maternal age (years)**			
15–24 (Ref.)		1.00	1.00
25–34		0.87(0.78–0.98)*	0.93(0.78–1.12)
35–49		0.93(0.82–1.05)	1.06(0.88–1.27)
**Marital status**			
Never in union (Ref.)		1.00	–
Married/living with partner		1.11(0.76–1.59)	–
Widowed/divorced/separated		1.37(0.88–2.13)	–
**Mother’s educational attainment**			
No education (Ref.)		1.00	1.00
Primary		0.88(0.76–1.03)	1.04(0.81–1.33)
Secondary or higher		0.74(0.63–0.86)***	0.96(0.80–1.16)
**Mother’s employment status**			
Not working (Ref.)		1.00	–
Currently working		1.04(0.94–1.15)	–
**Wealth quintile**			
Poor (Ref.)		1.00	1.00
Middle		0.92(0.79–1.06)	0.90(0.76–1.07)
Rich		0.70(0.60–0.81)***	0.68(0.55–0.84)***
**Place of residence**			
Urban (Ref.)		–	1.00
Rural		–	1.03(0.88–1.21)
**Region**			
North-central (Ref.)		–	1.00
North-east		–	0.95(0.76–1.19)
North-west		–	1.24(1.01–1.51)*
South-east		–	0.88(0.68–1.15)
South-south		–	0.84(0.62–1.15)
South-west		–	0.77(0.60–0.99)*

*p < 0.05; **p < 0.01; ***p < 0.001, *Ref.* reference category

## Results

### Distribution of the study population sample

The percentage distributions of the under-five mortality, maternal health insurance, healthcare services utilization and covariates are presented in Table 1. The results showed that 14.3% of the sampled 127,545 birth histories of childbearing women were children who died before age 5. The majority of the children were born to mothers who were not covered by health insurance (97.7%) and had adequate healthcare services utilization (56.5%). The largest proportion of children (58.7%) were those of mothers aged 35–49. An overwhelming majority of the children (91.9%) were born to mothers who reported being married or living together with partners. One-half of the children (50.0%) were born to mothers with no formal education. Over two-thirds of children (74.1%) had mothers who were currently working. The largest proportion of the children (47.5%) was born to mothers living in the poor household quintile. The mothers who reported to be rural residents had the majority of children (65.4%) in the sample. The proportion of children born to mothers in the sample ranged from 9.8 and 31.3% in the South-south and North-west, respectively. About 70% of the children were born to mothers who reported that distance to the health facility was not a big problem. Concerning decision-making, almost one-third of children (31.8%) were children of mothers who made joint decisions on their healthcare, while 72.2% were children of mothers whose partners made independent decisions on how their earnings are spent.

### Healthcare services utilisation by health insurance coverage

Table 2 presented the percentage distribution of the children whose mothers utilised healthcare services by health insurance coverage within 5 years before the survey. The results showed a significant relationship between healthcare services utilisation and health insurance. Over two-thirds (83.5%; *p* < 0.001) of the children whose mothers were covered by health insurance utilised adequate healthcare services, as compared with 16.5% who were not covered by health insurance.

### Maternal health insurance coverage, healthcare services utilisation and covariates associated with under-five mortality

Table 3 presents the bivariate relationship between the risk of U-5 M and the main explanatory variables, as well as covariates. The results showed that all the variables were significantly associated with U-5 M. For instance, the larger proportion of dead children was born to mothers who reported not being covered by health insurance (14.4%; *p* < 0.001) and those who had inadequate healthcare services (12.7%; *p* < 0.001). The results further showed that the highest proportion of dead children (15.7%; *p* < 0.001) were children of mothers aged 35–49 years; 14.4% for mothers currently married/living with partners; 18.5% for mothers with no formal education; 18.6 and 21.3% for mothers found in the poor wealth quintile households and North-west regions, respectively. Also, 16.7% of the dead children were of mothers whose husbands/partners made independent concerning their healthcare; and 15.3% for mothers who made sole decisions on how their earnings were spent. The larger proportions of dead children (29.4%; *p* < 0.001) were children of mothers who were not employed; 16.7% for mothers who were rural residents and 15.2% for those who reported that distance to the health facility was a big problem. Concerning decision-making, the largest proportion of the dead children (16.7%; *p* < 0.001 and 15.3%; *p* < 0.001) were children of mothers whose partners made independent decisions on their healthcare and those who enjoyed decision-making autonomy on how to spend their earnings, respectively.

### Risk factors of U-5 M: survival analysis

**Fig. 1:**
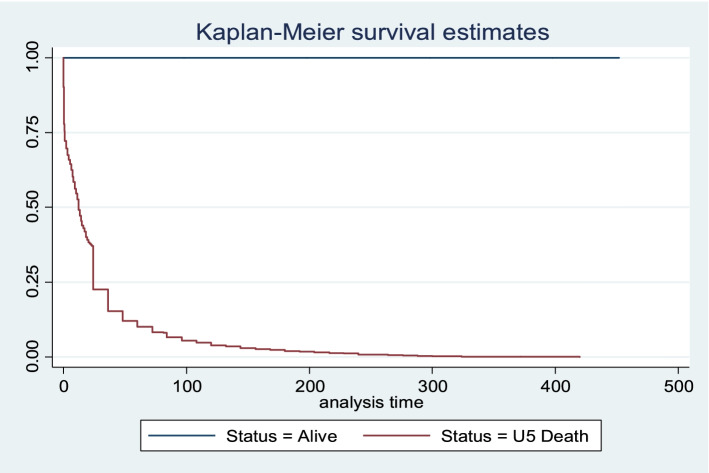
Child survival plot for children that died before reaching age five among all live-born children within 2013–2018

**Fig. 2:**
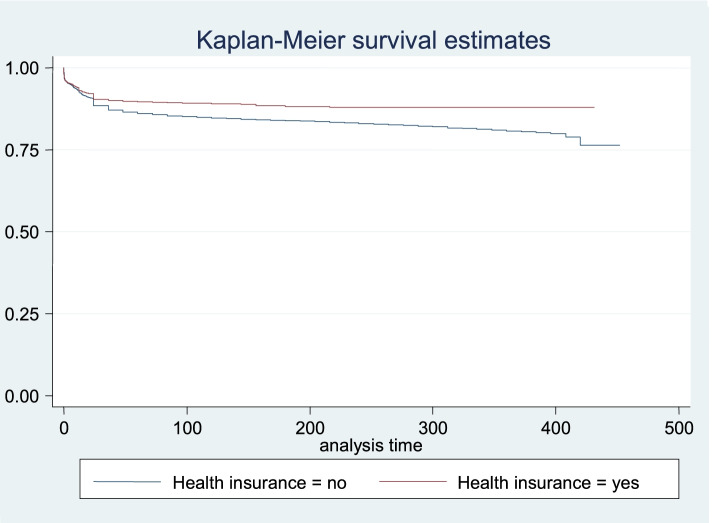
Child survival plot by health insurance for children that died before reaching age five among all live-born children within 2013–2018

**Fig. 3:**
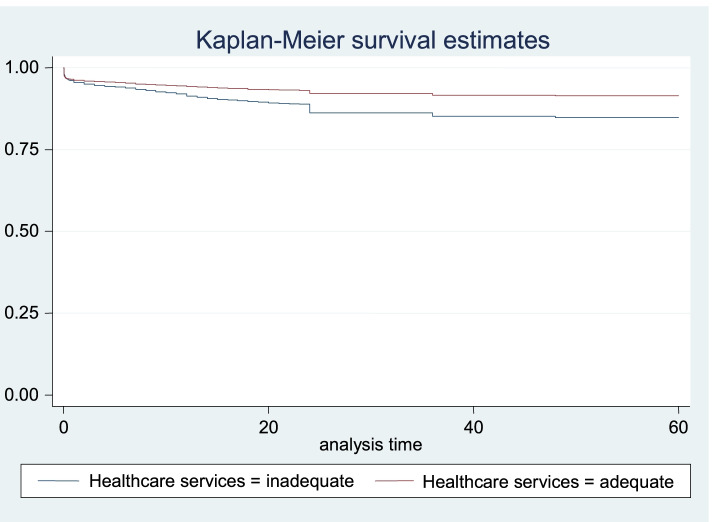
Child survival plot by health services utilisation for children that died before reaching age five among all live-born children within 2013–2018

The adjusted hazard results in Table 4, Models 1, 2 and 3 showed similar results for the main explanatory variables. In Table 4, Model 3, the risk of U-5 M was significantly reduced for children whose mothers reported to be covered by health insurance (HR: 0.62, CI: 0.46–0.88) and those who had adequate healthcare services utilisation (HR: 0.56, CI: 0.51–0.62), relative to those in the reference categories. Also, the risk of U-5 M was significantly reduced for children whose mothers reported that distance to health facilities was not a big problem (HR: 0.81, CI: 0.72–0.86) and those living in the rich wealth quintile households (HR: 0.68, CI: 0.55–0.84). Table 4, Model 3 further showed that in comparison with children of mothers in the reference category, the risk of U-5 M was significantly reduced among children of mothers residing in the South-west region of Nigeria (HR: 0.77, CI: 0.60–0.99) but increased for their counterparts in the North-west region (HR: 1.24, CI: 1.01–1.51). The description of the survival curves and functions as presented in Fig. 1 showed the child survival plot and duration of survival since birth for children that died within the first 5 years (0–59 months) among all live-born children. Also, Figs. 2 and 3 further described the mortality risks among children by maternal health insurance and healthcare services utilisation, respectively.

## Discussion

This study examined the impact of maternal health insurance coverage and adequate healthcare services utilisation on the risk of U-5 M in Nigeria. A consensus was observed with the previous studies on the significant relationship between maternal health insurance and healthcare services utilisation conducted in Nigeria [3], Ghana [47], Malawi [48] and other SSA countries [38, 49], our results revealed that maternal health insurance coverage had positive influence on maternal and child healthcare services utilisation. In line with the revealed low health insurance coverage of mothers, this study highlights the benefits of user fee exemption of healthcare services which is a crucial policy intervention for universal access to adequate maternal and child healthcare services, as well as improved health outcomes in Nigeria.

In agreement with previous studies on the significant association between maternal health insurance coverage and child’s health outcome in Ghana [50, 51] and other SSA countries [52], our findings further showed that the risk of under-five death was significantly lower among the children of mothers who were covered by health insurance and explained the importance of universal access to health insurance schemes in reducing the risks of U-5 M. As observed in previous studies [53, 54], this plausibly revealed the negative implications of mothers’ financial constraints in seeking healthcare, especially on their children’s health outcomes. This suggests the need for the expansion of health insurance schemes to every child under the age of 5 years, as well as accrediting health insurance schemes at the primary and private healthcare facilities to ensure the enrolment of disadvantaged people. No doubt, this becomes crucial to reducing U-5 M by having access to free and adequate healthcare services in Nigeria.

The study further revealed that the children of mothers who had access to adequate healthcare services utilisation were at lower risks of U-5 M. Our results are consistent with previous studies conducted in selected SSA countries [11, 55, 56]. This explains the fact that lack of access to adequate healthcare services, plausibly as a result of not being covered by health insurance by mothers is a contributory factor to the risk of child mortality. Consequently, the findings validate the observation that pregnant mothers who seek adequate healthcare could take postnatal care geared towards timely and appropriate health interventions for both maternal and childhood health problems [57].

Consistent with the previous studies that indicated the negative impact of long distances to the health facility on child health outcomes [58, 59]; this study revealed that long distances to the health facility significantly increased the risks of U-5 M. Plausibly, the location of health facilities at a far distance from home reduces the likelihood of seeking adequate healthcare services and increases the risk of child mortality [60]. Our findings further revealed significantly reduced risks of U-5 M among mothers who made joint decisions with partners on their healthcare and how their earnings are spent. This is in line with the previous observation that women’s involvement in decisions on their earnings could positively influence their healthcare services utilisation, hence improving maternal and child health outcomes [61]. Concerning the influence of a mother’s education on the risk of U-5 M, our findings are consistent with previous studies that mothers having secondary/tertiary educational attainment significantly reduced the risk of U-5 M compared with those with no formal education [62, 63]. Similarly, our findings corroborate previous studies in Nigeria and other selected SSA countries [64, 65], that living in the rich wealth quintile households is a protective factor against the risk of U-5 M mortality. This plausibly explains the fact that the mother’s education and household wealth might have operated through some healthcare policies including health insurance to reduce the risk of U-5 M. The findings of some regional variations in the risks of U-5 M could be attributed to the regional differentials in accessing healthcare services in Nigeria [10, 21].

Our findings have some policy implications since there might be considerable challenges in financing healthcare from both government agencies and private organisations in Nigeria. Therefore, to end preventable deaths of under 5 children, there is a need for the expansion of health insurance schemes to every under-five children, as well as accrediting such schemes at the primary and private healthcare facilities to ensure the enrolment of disadvantaged people located living away from well-equipped health facilities.

### Strengths and limitations

The main strengths of this study are the use of a national representative large sample of birth histories within 5 years before the survey and the adopted rigorous analytical procedures with weighted proportions. Also, the special focus on health insurance coverage from government agencies or private organisations and healthcare services utilisation marks a departure from previous studies in Nigeria.

This study has some limitations which include the use of cross-sectional DHS data which meant that cause-effect relationships could not be determined. In addition, the main explanatory variables and covariates were only temporal factors associated with child survival. There is a likelihood of most women reporting bias on health insurance coverage and healthcare services utilisation. Despite these limitations, the findings of this study are crucial for ending preventable deaths of newborns and children under the age of 5 years by ensuring universal health coverage and access to quality essential maternal and child healthcare services in Nigeria.

## Conclusion

In conclusion, there is low health insurance coverage among childbearing women. Also, maternal health insurance coverage and adequate healthcare services utilisation were found to be protective factors against the risk of U-5 M. More pragmatic policy and intervention programmes through universal maternal health insurance towards ending preventable deaths of children under the age of 5 years and ensuring universal access to quality healthcare services in Nigeria. This becomes imperative considering that the distance and cost barriers to seeking adequate healthcare services may be difficult for mothers to negotiate, hence the likelihood of experiencing a child’s death.

## Data Availability

The NDHS 2018 birth recode dataset was used for this study and is freely available from the DHS Program archive at https://www.dhsprogram.com/data/dataset.

## Abbreviations

U-5 M: Under-five mortality
SDGs: Sustainable Development Goals
HR: Hazard ratio
CIs: Confidence intervals
SSA: sub-Saharan Africa
NDHS: Nigeria Demographic and Health Survey
NHIS: National Health Insurance Scheme
HBM: Health Belief Model
DHS: Demographic and Health Survey
ANC: Antenatal Care
WHO: World Health Organisation

## References

[1] United Nations Inter-agency Group for Child Mortality Estimation. Levels & Trends in child mortality: report 2020, estimates developed by the United Nations inter-agency Group for Child Mortality Estimation, United Nations Children’s fund, New York. UN IGME 2020. URL: https://www.unicef.org/media/79371/file/UN-IGME-child-mortality-report-2020.pdf.

[2] Ng'anjo PS, Kiserud T, Kvåle G, Byskov J, Evjen-Olsen B, Michelo C (2013). Factors associated with health facility childbirth in districts of Kenya, Tanzania and Zambia: a population based survey. BMC Pregnancy Childbirth..

[3] Adewuyi EO, Auta A, Khanal V, Bamidele OD, Akuoko CP, Adefemi K (2018). Prevalence and factors associated with underutilization of antenatal care services in Nigeria: a comparative study of rural and urban residences based on the 2013 Nigeria demographic and health survey. PLoS One.

[4] Kuuire VZ, Kangmennaang J, Atuoye KN, Antabe R, Boamah SA, Vercillo S (2017). Timing and utilisation of antenatal care service in Nigeria and Malawi. Glob Public Health.

[5] World Health Organisation. Global consultation on integrated care for older people (ICOPE) – the path to universal health coverage: report of consultation meeting 23–25 October 2017 in Berlin, Germany. Geneva: WHO; 2018 (WHO/FWC/ALC/18.3). Licence: CC BY-NC-SA 3.0 IGO. https://apps.who.int/iris/bitstream/handle/10665/272863/WHO-FWC-ALC-18.3-eng.pdf

[6] Adesokun OO, Osemene KP, Ilori M, Ihekoronye RM (2020). Evaluation of enrollees’ perspectives on the operations of the National Health Insurance Scheme in Nigeria. J Health Med Sci.

[7] National Population Commission (NPC) [Nigeria] and ICF International. Nigeria Demographic and Health Survey 2013*.* Abuja, Nigeria, and Rockville, Maryland: NPC and ICF International; 2014. https://dhsprogram.com/pubs/pdf/FR293/FR293.pdf.

[8] Dixon J, Tenkorang EY, Luginaah IN, Kuuire VZ, Boateng GO (2014). National health insurance scheme enrolment and antenatal care among women in Ghana: is there any relationship?. Tropical Med Int Health.

[9] United Nations. World population prospects. Data booklet ST/ESA/SER.A/424**.** Department of Economic and Social Affairs**,** Population Division, UN 2019**.**

[10] National Population Commission (NPC) [Nigeria] and ICF International. Nigeria Demographic and Health Survey 2018. National Population Commission, Abuja, Nigeria, and ICF international 2019, Rockville. URL https://dhsprogram.com/pubs/pdf/FR359/FR359.pdf.

[11] Bado AR, Sathiya SA (2016). Women's education and health inequalities in under-five mortality in selected sub-Saharan African countries, 1990–2015. PLoS One.

[12] Yaya S, Uthman OA, Okonofua F, Bishwajit G (2019). Decomposing the rural-urban gap in the factors of under-five mortality in sub-Saharan Africa? Evidence from 35 countries. BMC Public Health.

[13] Imo CK, Ukoji UV (2020). Maternal socio-demographic factors vs antenatal care utilization and under-five mortality in Nigeria. Gender Behaviour.

[14] Fasina F, Oni G, Azuh D, Oduaran A (2020). Impact of mothers’ socio-demographic factors and antenatal clinic attendance on neonatal mortality in Nigeria. Cogent Soc Sci.

[15] Azuh DE, Chinedu S, Samuel OW, Azuh A, Joshua G, Amoo EO (2019). Factors influencing the survival of under-five children among women visiting government health care facility in semi-urban communities in Nigeria. Cogent Arts Humanit.

[16] Adedokun ST, Adekanmbi VT, Uthman OA, Lilford RJ (2017). Contextual factors associated with health care service utilization for children with acute childhood illnesses in Nigeria. PLoS One.

[17] Adedini SA, Odimegwu C, Imasiku EN, Ononokpono DN (2015). Ethnic differentials in under-five mortality in Nigeria. Ethn Health.

[18] Gbadebo BM, Bamiwuye SO, Bisiriyu LA (2018). Family type, ethnicity and under-five mortality in Nigeria. Afr Popul Stud.

[19] Adebowale SA, Morakinyo OM, Ana GR (2017). Housing materials as predictors of under-five mortality in Nigeria: evidence from 2013 demographic and health survey. BMC Pediatr.

[20] Samuel OW, Oni GA, KC S, Wurzer M, Akinyemi AI. Household use of solid fuel for cooking and under-five mortality in Nigeria. Afr Popul Stud 2018; 32(1): 4034–42. 10.11564/32-1-1175.

[21] Imo CK, De Wet-Billings N. Socio-ecological determinants of under-five mortality in Nigeria: exploring the roles of neighbourhood poverty and use of solid cooking fuel. J Biosoc Sci. 2021:1–3. 10.1017/S0021932021000614. 10.1017/S0021932021000614 34743767

[22] Otekunrin OA, Ayinde IA, Sanusi RA, Onabanjo OO, Ariyo O (2022). Dietary diversity, environment and health-related factors of under-five children: evidence from cassava commercialization households in rural south-West Nigeria. Environ Sci Pollut Res.

[23] Becker MH (1974). The health belief model and personal health behaviour. Health Educ.

[24] Abraham C, Sheeran P. The health belief model. Predicting health behaviour: Research and practice with social cognition models 2015; 2:30–55.

[25] Strecher VJ, Rosenstock IM. The health belief model. Cambridge handbook of psychology. Camb Handb Psychol Health Med. 1997:113–7.

[26] Adhikari RP, Shrestha ML, Satinsky EN, Upadhaya N (2021). Trends in and determinants of visiting private health facilities for maternal and child health care in Nepal: comparison of three Nepal demographic health surveys, 2006, 2011, and 2016. BMC Pregnancy Childbirth..

[27] Janz NK, Becker MH (1984). The health belief model: a decade later. Health Educ Q.

[28] Abraham C, Sheeran P, Conner M, Norman P (2005). The health belief model. Predicting health behaviour: research and practice with social cognition models.

[29] Adugna MB, Nabbouh F, Shehata S, Ghahari S (2020). Barriers and facilitators to healthcare access for children with disabilities in low and middle income sub-Saharan African countries: a scoping review. BMC Health Serv Res.

[30] World Health Organization (2013). World health statistics.

[31] Atuoye KN, Dixon J, Rishworth A, Galaa SZ (2015). Can she make it? Transportation barriers to accessing maternal and child health care services in rural Ghana. BMC Health Serv Res.

[32] Da Costa D, Ireland K (2013). Perceived benefits and barriers to leisure-time physical activity during pregnancy in previously inactive and active women. Women Health.

[33] Chakraborty N, Islam A, Chowdhury RI, Bari W (2002). Utilization of postnatal care in Bangladesh: evidence from a longitudinal study. Health Soc Care Community.

[34] Kabir M, Iliyasu Z, Abubakar IS, Sani AA (2005). Determinants of utilization of antenatal care services in Kumbotso village, Northern Nigeria. Trop Doct.

[35] Engl E, Kretschmer S, Jain M, Sharma S, Prasad R, Ramesh BM (2019). Categorizing and assessing comprehensive drivers of provider behavior for optimizing quality of health care. PLoS One.

[36] Rosenstock IM, Strecher VJ, Becker MH (1988). Social learning theory and the health belief model. Health Educ Q.

[37] Ajzen I, Fishbein M. Understanding attitudes and predicting social behaviour. Prentice-Hall, Inc., Englewood Cliffs, New Jersey 07632, 1980.

[38] Ahinkorah BO, Budu E, Seidu AA, Agbaglo E, Adu C, Ameyaw EK, Ampomah IG, Archer AG, Kissah-Korsah K, Yaya S (2021). Barriers to healthcare access and healthcare seeking for childhood illnesses among childbearing women in sub-Saharan Africa: a multilevel modelling of demographic and health surveys. PLoS One.

[39] Sripad P, Kirk K, Adoyi G, Dempsey A, Ishaku S, Warren CE (2019). Exploring survivor perceptions of pre-eclampsia and eclampsia in Nigeria through the health belief model. BMC Pregnancy Childbirth.

[40] You D, Hug L, Ejdemyr S, Idele P, Hogan D, Mathers C (2015). Global, regional, and national levels and trends in under-5 mortality between 1990 and 2015, with scenario-based projections to 2030: a systematic analysis by the UN inter-agency Group for Child Mortality Estimation. Lancet.

[41] World Health Organization. Delivering quality health services: a global imperative for universal health coverage. Geneva: World Health Organization, Organisation for Economic Co-operation and Development, and the World Bank; 2018. Licence: CC BY-NC-SA 3.0 IGO.

[42] World Health Organization. WHO antenatal care randomized trial: manual for the implementation of the new model. WHO 2002, Geneva. URL https://apps.who.int/iris/handle/10665/42513

[43] World Health Organization. WHO recommendations on antenatal care for a positive pregnancy experience, 2016 Geneva. URL: http://apps.who.int/iris/bitstream/10665/250796/1/9789241549912-eng.pdf. 28079998

[44] Nigatu D, Gebremariam A, Abera M, Setegn T, Deribe K. (2014). Factors associated with women’s autonomy regarding maternal and child health care utilization in bale zone: a community based cross-sectional study. BMC Womens Health 2014; 14:79. 10.1186/1472-6874-14-79. 10.1186/1472-6874-14-79 PMC4094397 24990689

[45] Osamor PE, Grady C (2016). Women's autonomy in health care decision-making in developing countries: a synthesis of the literature. Int J Women's Health.

[46] Cox DR (1972). Regression models and life-tables. J R Stat Soc.

[47] Twum P, Qi J, Aurelie KK, Xu L (2018). Effectiveness of a free maternal healthcare programme under the National Health Insurance Scheme on skilled care: evidence from a cross-sectional study in two districts in Ghana. BMJ Open.

[48] Manthalu G, Yi D, Farrar S, Nkhoma D (2016). The effect of user fee exemption on the utilization of maternal health care at mission health facilities in Malawi. Health Policy Plan.

[49] Kanyangarara M, Munos MK, Walker N (2017). Quality of antenatal care service provision in health facilities across sub-Saharan Africa: evidence from nationally representative health facility assessments. J Glob Health.

[50] Anaba EA, Abuosi AA, Azilaku JC, Nkrumah J (2020). Association between health insurance membership and anaemia among children under-five years. Evidence from Ghana. PLoS ONE.

[51] Bosomprah S, Ragno PL, Gros C, Banskota H (2015). Health insurance and maternal, newborn services utilisation and under-five mortality. Arch Public Health.

[52] Simmons RA, Anthopolos R, O’Meara WP (2021). Effect of health systems context on infant and child mortality in sub-Saharan Africa from 1995 to 2015, a longitudinal cohort analysis. Sci Rep.

[53] Mukonka PS, Mukwato PK, Kwaleyela CN, Mweemba O, Maimbolwa M (2018). Household factors associated with use of postnatal care services. Afr J Midwifery Womens Health.

[54] Stack RJ, Meredith A (2018). The impact of financial hardship on single parents: an exploration of the journey from social distress to seeking help. J Fam Econ Iss.

[55] Dominic A, Ogundipe A, Ogundipe O (2019). Determinants of women access to healthcare services in sub-Saharan Africa. Open Public Health J.

[56] Abreha SK, Zereyesus YA (2021). Women’s empowerment and infant and child health status in sub-Saharan Africa: a systematic review. Matern Child Health J.

[57] Getachew Y, Bekele S (2016). Survival analysis of under-five mortality of children and its associated risk factors in Ethiopia. J Biosens Bioelectron.

[58] Boettiger DC, Treleaven E, Kayentao K, Guindo M, Coumaré M, Johnson AD (2021). Household factors and under-five mortality in Bankass, Mali: results from a cross-sectional survey. BMC Public Health.

[59] Karra M, Fink G, Canning D (2017). Facility distance and child mortality: a multicountry study of health facility access, service utilization, and child health outcomes. Int J Epidemiol.

[60] Seidu AA (2020). Mixed effects analysis of factors associated with barriers to accessing healthcare among women in sub-Saharan Africa: insights from demographic and health surveys. PLoS One.

[61] Annan J, Donald A, Goldstein M, Martinez PG, Koolwal G (2021). Taking power: Women’s empowerment and household well-being in sub-Saharan Africa. World Dev.

[62] Van Malderen C, Amouzou A, Barros AJD, Masquelier B, Oyen HV, Speybroeck N (2019). Socio-economic factors contributing to under-five mortality in sub-Saharan Africa: a decomposition analysis. BMC Public Health.

[63] Yaya S, Bishwajit G, Okonofua F, Uthman OA (2018). Under five mortality patterns and associated maternal risk factors in sub-Saharan Africa: a multi-country analysis. PLoS One.

[64] Ekholuenetale M, Wegbom AI, Tudeme G (2020). Household factors associated with infant and under-five mortality in sub-Saharan Africa countries. Int J Child Care Educ Policy.

[65] Yaya S, Ekholuenetale M, Tudeme G, Vaibhav S, Bishwajit G, Kadio B (2017). Prevalence and determinants of childhood mortality in Nigeria. BMC Public Health.

